# Accuracy of Parental Reporting of Preschoolers’ Dietary Intake Using an Online Self-Administered 24-h Recall

**DOI:** 10.3390/nu10080987

**Published:** 2018-07-29

**Authors:** Angela Wallace, Sharon I. Kirkpatrick, Gerarda Darlington, Jess Haines

**Affiliations:** 1 Family Relations and Applied Nutrition, University of Guelph, Guelph, ON N1G 2W1, Canada; jhaines@uoguelph.ca; 2 School of Public Health and Health Systems, University of Waterloo, Waterloo, ON N2L 3G1, Canada; sharon.kirkpatrick@uwaterloo.ca; 3 Department of Mathematics and Statistics, University of Guelph, Guelph, ON N1G 2W1, Canada; gdarling@uoguelph.ca

**Keywords:** dietary assessment, parent report, nutrition assessment, preschool nutrition, child nutrition, 24-h recalls, online dietary assessment

## Abstract

Parents are typically relied upon to report young children’s dietary intake. However, there has been limited research assessing the accuracy of such reports captured using novel dietary assessment tools. The purpose of the current study was to assess the validity of the web-based Automated Self-Administered 24-h Dietary Assessment Tool (ASA24-Canada) for capturing dietary intake among children aged two-five years (*n* = 40), using parental proxy reporting. The study was conducted in a daycare setting, allowing for standardization of foods and drinks offered and direct observation of intake. Parental-reported intake was compared to true intake for lunch and dinner, as well as an afternoon snack, on a single day. Each eating occasion, including plate waste, was unobtrusively documented. Parents were not present for lunch or the afternoon snack, but joined their children at the daycare centre for the dinner meal. The following day, parents reported their children’s intake from the previous 24-h period using ASA24-Canada. For the eating occasions assessed, parents reported exact or close matches for 79.2% (82.3% for lunch, 81.2% for the snack, and 77.4% for dinner) of the foods and beverages truly consumed by children. Estimates of intake for energy and macronutrients examined (carbohydrates, fat, and protein) based on parental reports were higher than those based on true (observed) intake. Our findings suggest that parents are able to report what their preschool children eat and drink relatively accurately. However, the accuracy of portion size estimates is low. Strategies to enhance portion size reporting are needed to improve parental proxy reporting.

## 1. Introduction

The accurate assessment of dietary intake is critical in efforts to understand influences on eating patterns, to assess relationships between diet and health outcomes, and for evaluating the outcomes of dietary interventions [1]. Such efforts are important within the context of childhood as food preferences established early in life have been shown to influence eating patterns into adulthood [2,3], with implications for later health and disease risk. Therefore, identifying ways to accurately assess young children’s dietary intake will benefit clinicians, researchers, and policy makers in their efforts to support the development of healthy dietary behaviours in early childhood [2].

Existing evidence suggests that the interview-administered 24-h recall is the most accurate method to assess children’s dietary intake [4]. Parents are typically relied upon as proxy reporters for capturing dietary intake [5], because young children, i.e., those eight years of age and younger, generally have immature cognitive abilities (which affect their ability to estimate portion sizes, for example), incomplete concepts of time, and potentially limited knowledge of food names and preparation methods [6,7]. For example, the National Health and Nutrition Examination Survey (NHANES) used to assess health and nutrition status of adults and children in the United States uses proxy reporting for children under age five and assisted reporting for children aged 6–11 years [4]. Similar procedures are used for the nutrition-focused cycles of the Canadian Community Health Survey (CCHS) [8].

However, 24-h recalls have traditionally required highly trained staff to conduct interviews and code intake data using specialized software, making the method expensive and burdensome [9]. Innovations in dietary assessment and technology have resulted in the development of automated 24-h recalls intended to enable researchers and clinicians to efficiently collect comprehensive dietary data [9,10]. For example, the Automated Self-Administered 24-h Dietary Assessment Tool (ASA24) is a web-based tool developed by the U.S. National Cancer Institute (Rockville, MD, United States) and adapted to Canada (ASA24-Canada) to enable the collection of multiple, automatically-coded, self-administered recalls in a range of settings [10]. 

Few studies have assessed the accuracy of 24-h recalls relying upon parental-proxy reporting to capture the intake of preschool-aged children [11,12,13,14,15,16,17]. Many children of this age spend a relatively large proportion of time within child care settings. In Canada, approximately 46% [18] of parents use some form of childcare, and 61% of children under five are in some form of organized care in the United States [19]. These statistics suggest that many eating occasions occur in settings within which parents are not present. Therefore, it is important to assess parents’ ability to accurately report their children’s intake, even for eating occasions for which they may not be present. The aim of this study was to assess parents’ ability to accurately report their preschool child’s true dietary intake using ASA24-Canada for three separate eating occasions occurring in a childcare setting.

## 2. Methods

This study took place at the Child Care and Learning Centre at the University of Guelph, Canada. For simplicity, the term Child Care Centre will be used throughout the manuscript to describe this location. The University of Guelph Research Ethics Board (REB) reviewed and approved the study protocol (REB # 15JN028).

### 2.1. Participants 

Parents and children were recruited at the Child Care Centre using in-person information sessions, preceded by an information letter email sent to all parents. Children eligible to participate were two to five years of age, attended the Child Care Centre, and had no parent-reported dietary restrictions that would be difficult to accommodate (i.e., allergy to a menu item). In addition, eligible dyads included parents who were able to speak, read, and write in English. Dyads were ineligible if parents had extensive nutrition training, e.g., a nutrition degree. In cases where eligible parents had multiple children attending the Child Care Centre, the child with the closest birth date to the study start date was selected. Approximately 100 parents were approached to participate in the study, and 42 provided written informed consent. Recalls for two children were excluded, as described below; thus, the final sample was 40 parent–child dyads. 

Data were collected on two consecutive days from November 2015 to March 2016, based on a protocol previously used to assess the validity of ASA24 for capturing true intake among adults [20]. On day one child participants were unobtrusively observed during lunch and afternoon snack at the Child Care Centre. The Child Care Centre provides children with lunch and snacks for the day based on a four-week cycled menu. Lunch offerings on study days included salmon, brown rice, broccoli, apple slices, and milk. Snack offerings included whole wheat crackers and cheddar cheese. The same foods and beverages were offered to all children at the Centre, regardless of participation. Centre meals are typically served family style (i.e., from communal containers from which staff serve children), but for the purpose of this study, items served to participating children were weighed (as described below using My Weigh WR-12K waterproof scale, Canada) and pre-plated. If a participating child asked for seconds, staff discreetly placed the additional food on a plate for unobtrusive weighing by a research assistant before it was given to the child. For both lunch and snack, all parents could view menus posted at the centre and on the centre website, as per regular Centre procedures. In addition, parents had access to an app called Hi Mama (Hi Mama Inc. 2016, Toronto, ON, Canada), which was being tested within the Child Care Centre and provided parents with information on their child’s eating, including the foods served and consumed, and washroom and sleeping patterns throughout the day.

The centre does not typically serve dinner to children; however, as part of the study participating families were invited to dinner and unobtrusively observed. Parents, and in some cases other family members (e.g., siblings), joined the child participant at the Child Care Centre for dinner, which took place in a large classroom transformed into a dining area with tables, chairs, and soft music. Dinner offerings included pasta with beans or ground meat, mixed green salad, garlic bread, fruit salad, milk, water, juice, and cookies. Food and beverages were served buffet style, with one table specifically for the child participants and a second for parents and other family members. A parent from each dyad was invited to select and serve food and beverage items for the participating child from the child buffet, and then to do the same for themselves and other family members from the family buffet. 

All food and beverage items for the lunch, snack, and dinner eating occasions were weighed prior to serving using a standardized weighing protocol developed for a similar study that analysed the validity of ASA24 among adults [20]. Trained research assistants weighed each food/beverage item twice and if the two weights varied by more than 1 g, a third measurement was taken and the two closest weights were averaged. All food waste was collected and weighed using the same protocol. Lunch and afternoon snack items and single-serve items on the dinner buffet were weighed individually prior to serving. Communal containers on the dinner buffet were weighed prior to and after each parent selected items for their child to determine the amount taken. The amount consumed (true intake) for each item at each meal was calculated by subtracting plate waste weight from the amount served to each child. 

On day two, parents returned to the Child Care Centre during regular “drop off” hours and completed two online surveys: a brief demographic questionnaire and an unannounced 24-h recall using ASA24-Canada (parents were told in advance they would be asked to complete a survey to learn about their child’s food habits). The study coordinator (AW) instructed participants to open their email, where they would find two survey links, one to a demographic survey and the second to ASA24-Canada. ASA24 assumes self-administration, but the study coordinator prompted parents to report on behalf of their children rather than themselves. Nine parents were unable to complete the surveys at the centre during the morning drop off due to medical appointments or work commitments and completed them from another location (e.g., work or home) on the appointed day (i.e., the day after the meal observation). After completing the surveys, participants received a $25 grocery store gift card to thank them for their time. 

### 2.2. Data Management and Coding

#### 2.2.1. True Intake

Each item offered was coded using the Canadian Nutrient File (CNF) [21]. The corresponding nutrient values were applied to each child’s true intake (amount consumed) for each food and beverage, allowing estimation of energy and nutrient content for each eating occasion and for all three eating occasions together. 

#### 2.2.2. Reported Intake

Protocols and procedure were adapted for the purposes of this study from a similar validation study conducted with adults using ASA24 [22]. When completing ASA24-Cananda, parents were prompted to recall all food consumed in the previous 24 h on behalf of their child. However, for the purpose of analyses, only meals and snacks consumed at the Child Care Centre were analysed. Meals that were not observed, e.g., breakfast and evening snack, were removed from the ASA24-Canada data after review of reported eating occasions by name and time to verify those that aligned with the observed meals. During this process, two recalls were removed from analyses; one parent notified the study coordinator that she mistakenly completed the recall for herself instead of her child and one parent provided a recall that included no foods from the observation day, suggesting the recall was completed for the incorrect time frame.

Subsequently, a list of all food and beverages reported by parents for the study meals was created and sorted by eating occasion. This list was independently reviewed by two Registered Dietitians, the first author and a second dietitian who was not otherwise involved in the research. Each item was compared to the menu (i.e., items served) to determine whether each was an exact, close, or far match. For example, apples were served during lunch; therefore, apple was considered an exact match, and a similar single serving of fruit such as a pear was considered a close match. If the parent marked down an uncertainty of the type of fruit or a generalization, like fruit salad, the item was considered a far match. One instance in which the two reviewers disagreed in terms of whether a food was an exact, close, or far match was discussed by the full study team in order to come to a consensus. 

Through this procedure (which was conducted with blinding to the true intake data), a “match key” was developed and then applied to compare the true and recalled intakes for each child. This resulted in the identification of matches (items consumed and reported), exclusions (items consumed but not reported) and intrusions (items reported but not consumed). Intrusions were identified as internal (offered at one of the eating occasions) or external (not offered) confabulations. This procedure was designed based on previous work from Kirkpatrick and colleagues [20] and Baxter and colleagues [21]. The procedure was conducted for all study meal occasions and reviewed for accuracy by a dietitian who was not otherwise involved.

## 3. Statistical Analyses 

Analyses were conducted using SPSS, version 23 (IBM Analytics, Armonk, NY, USA). Frequencies were calculated to determine the proportion of food and beverages truly consumed for which matches were reported, as well as the proportion of exclusions and the most common exclusions, by meal and overall. Additionally, the mean number of intrusions (items not eaten by child but reported by parent) was estimated. Analyses of portion size accuracy were conducted for each meal occasion separately. It was hypothesized that differences in portion might exist between meals due to the presence or absence of the parent during that meal. 

The majority of parents (*n* = 29) recalled their child’s intake for all study meals (lunch, afternoon snack, dinner); however, 11 parents reported intake only for the dinner meal at which they were present. In order to analyse and assess the proportion of inclusions and exclusions, we used the full sample of 40 participants and did not consider the 11 participants missing meal reports as exclusions.

Paired *t*-tests were used to examine differences between energy and macronutrient intakes based on true versus recalled intakes for each meal occasion. Paired *t*-tests were also used to determine difference between portion intakes based on true versus reported intake for each meal occasion and for each food offering. For portion size, only those items for which exact and close matches were reported could be analysed. 

## 4. Results

Key characteristics of the parent and child participants are outlined in Table 1. Among the 40 dyads, 84% (*n* = 33) of the participating parents were mothers. The majority of families (65%) had an average household income ranging from $100,000–150,000 or more. Additionally, 89% of parents reported having a university graduate degree or having postgraduate training. The majority (86%) of the parent reporters identified as white; however, 22% percent of parents were born outside Canada. 

An overview of matches by study meals is shown in Table 2. The mean proportion of food and beverages consumed for which parents reported matches (exact, close and far) were 82.3% for lunch, 82.1% for snack, and 77.4% for dinner (Table 2). The proportion of exclusions across all observed meals was 20.8% (Table 2). The highest proportion of exclusions was observed for the dinner meal (22.6%; Table 2). The most frequent exclusions (Table 3) were additions to meals (e.g., salad dressing) or items from multi-component dishes (e.g., items from fruit salad). The majority of the exclusions (except for the apple) came from the dinner meal. The mean number of intrusions across all observed meals was 1.2 (Table 2). Intrusions were least common for the dinner meal for which parents served their children (Table 2).

When including the children whose parents did not report lunch and snack (for whom the proportion of matches for a given meal is zero and the proportion of exclusions is 100), matches decreased to 75.3% and 66.3%, respectively.

Estimates of intake of energy and most macronutrients were higher based on parent-reported food and beverage consumption than estimates based on true intake (Table 4). 

Reported portion sizes were larger than true portion sizes across all meal occasions (Table 5). Results suggest that there was a larger difference in mean portion estimation between true and reported intake for the lunch meal occasion compared to dinner and snack (Table 5). When comparing portion sizes by food offerings our results suggest that parents overestimated their children’s intake for most items (Table 6); exceptions were crackers and cookies. 

## 5. Discussion

To our knowledge, this is the first study to use direct observation to examine parents’ ability to report their preschool children’s intake using an online 24-h recall. We found that parents were able to report the food and beverages consumed relatively accurately, capturing about four out of five of the items actually consumed. However, we also found that one in four parents reported only the meal occasion for which they were present with their child. We are unable to determine whether parents did not understand the instructions to report their child’s intake for the entire day or whether they felt unable to report what their child ate and drank when they were not present. Though likely an artefact of our study design, the omission of meals at which parents were absent highlights the importance of clear instructions for tasks such as reporting another person’ intake.

The number of intrusions was lower for the dinner meal as opposed to lunch and the snack. This is likely because parents served their children their dinner meal (whereas they were not present at lunch or snack) and presumably were better able to identify exactly what was consumed as compared to the other eating occasions. On the other hand, the number of exclusions was higher for the dinner meal as compared to lunch and snack offerings. This may be due to the fact that the dinner meal consisted of more additions, e.g., salad dressing, and multi-component items, e.g., salad, whereas the lunch meal consisted of single entity items such as salmon, broccoli and brown rice. In their study to assess the validity of ASA24 for use with adults, Kirkpatrick and colleagues [20] also found that common exclusions were ingredients in or additions to multi-component dishes. In contrast, the number of intrusions was higher for the eating occasions for which parents were not present and these intrusions tended to be internal confabulations (offered but not consumed by the child). This suggests that parents had some familiarity with what was served in the child care setting and made educated guesses about what their children consumed. Indeed, parents had reasonably good access to menu information visible in multiple locations and the opportunity to access information on the child’s eating patterns, i.e., the particular foods their child ate, using the Hi Mama app, although amount served and consumed was not reported (Hi Mama Inc. 2016, Toronto, ON, Canada). The extent to which this information was accessed was not examined; thus, the influence on our results is unknown. 

Although the lunch and snack eating occasions did not necessarily include any items that would be viewed as unhealthy by parents, at dinner, we offered both fruit salad and cookies and a choice of milk, juice, and water. We did not find any evidence of parents modifying selections based on perceived healthfulness of items, as many children consumed both cookies and juice during the dinner meal. In addition, based on our exclusion analysis, parents did not appear to exclude the items typically viewed as unhealthy. However, the cookie was one of the only items that were not overestimated by parents, which may suggest that parents were more conscious of the amount their child consumed of less healthful foods as compared to more healthful foods. Similar results were found for juice; although juice intake was overestimated by parents, the difference between actual and reported intake was not statistically significant. In addition to possibly being perceived as less healthful by parents, the cookie and juice were also single-serve items. Thus, it is possible that parents were better able to report their child’s intake of these single serve items as compared to mixed dishes or foods served in communal containers.

It should be noted that the dinner meal in which parents were present lead to less significant overestimation (see Table 6), suggesting that parents do a better job estimating food and beverage intake when they are observing mealtime. We do see a significant difference in milk overestimation at both lunch and dinner, suggesting parents have difficulty reporting milk quantities. These findings are similar to the Canadian Community Health Survey (CCHS) [23] and previous work done by Fisher and colleagues [24], which suggests milk as a major source of overestimation for proxy report on behalf of toddlers.

We found that estimated energy and macronutrient intakes based on parents’ reports were higher than true intakes. These results are comparable to previous studies using parental report, which also demonstrate overestimation in total energy intake ranging from 7–29% [24,25,26]. Overestimation of energy and nutrient intakes is consistent with our observed overestimation of portion sizes by parents. Similar errors in portion size estimation have been found in previous studies with young children using proxy reporting [11,15,24]. The Canadian Community Health Survey (CCHS), which used interview administered 24-h recalls, found that approximately 30% of parents over reported the intake of their two-three year old children. As children age, the tendency for over-reporting diminished [23]. Previous research has also found that child age is inversely associated with portion size estimation error, with improved parent reporting in school-aged children compared to preschool-aged children [24]. This may be a result of portion sizes among older children becoming similar (almost mimicking) to those consumed by adults [25]. As well, older children may be able to provide some insights to parents in terms of how much they consumed. Portion size estimation remains a challenge in dietary assessment across age groups [26]. Possible strategies to improve estimation could include providing parents with 3D food and beverage visuals and training on portion size estimation prior to self-administration of the 24-h recall [24,27].

The findings of this study should be interpreted in light of several considerations. Importantly, parents had opportunities to familiarize themselves with the Child Care Centre menu and were likely to have a general idea of the types of foods and beverages offered, even if they weren’t aware of the specific items offered for lunch and snack on the recalled day. Thus, our findings may not be generalizable to parents who are not provided with information on their child’s food offerings within a child care setting. Further, participation in the study itself may have impacted reporting accuracy; parents knew that their children were participating in a study about eating and may have paid special attention to the menu offerings for the study days. Additionally, though efforts were made to ensure that study procedures were carried out in an unobtrusive manner, children may have noticed increased attention to their food and beverages. Given their ages, it is unlikely that they were able to pass along information to their parents that would have enhanced the accuracy of reporting. 

The study was conducted using a relatively high-income and highly-educated sample and the findings may not be generalizable to populations in which literacy or computer literacy may be limited. The majority of families in our sample (65%) had incomes ranging between $100,000 and $150,000 per household, whereas the majority of Canadian families (60.8%) have an average income below $100,000 [28]. Additionally, three in four parents had completed postgraduate training, compared to 30% for Canada more broadly. [29]. Similarly, the majority of our sample identified as “white” (86%). Future research should assess the accuracy of ASA24 for use among more diverse socioeconomic and racial/ethnic families with young children. The study aimed to reflect “real-life” settings where parents may be asked to recall their child’s intake during meals they were not present for (i.e., when child is in daycare). Although parents had access to the menu and the Hi Mama app (Hi Mama Inc. 2016, Toronto, ON, Canada), they did not have access to portion sizes served and consumed. This lack of information could have potentially contributed to the larger overestimation observed during the lunch and snack meal occasions.

In conclusion, we found that parents of preschool children were able to report the food and beverages that their child consumed, including for two eating occasions that occurred outside of their presence, with reasonable accuracy. However, to improve parental proxy reporting continued strategies to enhance portion size reporting are needed. 

## Figures and Tables

**Table 1:** Characteristics of the parent–child dyad participants (*n* = 40) of the observation feeding study.

	*N* (%)
**Parent Characteristics**	
**Sex (*n* = 40)**	
Father	7 (17.5%)
Mother	33 (82.5%)
**Household Income (*n* = 37)**	
10,000–49,999	4 (10.8%)
50,000–99,999	9 (24.3%)
100,000–150,000+	24 (64.8%)
**Level of Education (*n* = 37)**	
College graduate	4 (10.8%)
University graduate	6 (16.2%)
Post graduate training or degree	27 (72.9%)
**Race-Ethnicity (*n* = 37)**	
White	32 (86.4%)
Other (including Black, Latin American, South Asian, and Chinese)	5 (13.6%)
**Child Characteristics**	
**Sex**	
**Male**	12 (30.0%)
**Female**	28 (70.0%)
**Age years**	
2.0–2.9	7 (18.2%)
3.0–3.9	16 (40.6%)
4.0–5.9	17 (42.5%)

**Table 2:** Mean proportion of exact, close, and far matches and exclusions and number of intrusions reported by parents (*n* = 40) using ASA24-Canada for each meal occasion, in relation to true (observed) intakes. ^1^

Matches	Lunch (Parent not Present) (*n* = 29)	Afternoon Snack (Parent not Present) (*n* = 27)	Dinner (Parent Present) (*n* = 40)
Exact Matches (%)	61.6	78.8	57.3
Close Matches (%)	15.3	2.4	17.5
Far Matches (%)	5.4	0.0	2.6
All Matches Combined (%)	82.3	81.2	77.4
Exclusions (%)	17.7	18.8	22.6
Mean intrusions (*n*)	0.8	0.9	0.3
Mean items reported (*n*)	5.5	3.5	10.0

^1^ Exclusions were identified as items consumed but not reported. Intrusions were identified as items reported but not consumed).

**Table 3:** Counts of most common exclusions for all meals reported by parents (*n* = 40) using ASA24-Canada in relation to true (observed) intakes.

Items	Count	Percent
Apple	7	5.0
Garlic Bread	11	7.9
Maple Balsamic Dressing	7	5.0
Mandarin Oranges	12	8.6
Pineapple	11	7.9
Tomato Sauce with Beans	8	5.7
Red Peppers	8	5.7

**Table 4:** Energy and macronutrient differences between true and reported intake during each meal occasion (lunch, snack, and dinner).

Nutrient	True Intake	Reported Intake	Difference between True and Reported (95% CI)
Lunch Meal (*n* = 29)			
Energy	235.39	358.50	−123.11 (−177.10, −69.13)
Carbohydrates	25.61	40.43	−14.82 (−21.91, −7.74)
Protein	15.07	7.10	7.97 (4.97, 11.15)
Fat	8.22	12.14	−3.91 (−6.21, −1.62)
Snack Meal (*n* = 27)			
Energy	109.94	156.22	−46.28 (−84.01, −8.57)
Carbohydrates	10.86	8.99	1.87 (−0.94, 4.68)
Protein	4.34	7.17	−2.82 (−4.82, −0.83)
Fat	5.40	10.83	−5.42 (−8.41, −2.44)
Dinner Meal (*n* = 40)			
Energy	292.68	372.05	−79.36 (−135.41, 23.32)
Carbohydrates	48.48	59.53	−11.05 (−20.32, −1.79)
Protein	7.43	12.29	−4.85 (−7.06, −2.65)
Fat	8.08	9.84	−1.86 (−3.67, 0.15)

Bolded values represent significance at *p* < 0.05, confidence interval does not include 1.

**Table 5:** Mean portion intake (g) based on true and recalled intakes by meal occasion.

Portions by Meal Occasion Models (Paired *t*-Test)	True Intake	Recalled Intake	Difference between True and Reported (95% CI)
Lunch (true grams—recalled grams) *n* = 29	288.5	445.5	**−156.9 (−234.1, −79.6)**
Afternoon Snack (true grams—recalled grams) *n* = 29	103.4	187.9	**−84.5 (−130.7, −38.3)**
Dinner (true grams—recalled grams) *n* = 40	341.3	411.4	**−70.1 (−123.7, −16.5)**

Bolded values represent significance at *p* < 0.05, confidence interval does not include 1.

**Table 6:** Average amounts (g) of true and reported intake by food offering.

Food	True	Reported	Difference between True and Reported (95% CI)
**Lunch Meal Offerings (true grams–recalled grams)**			
Salmon	47.2	89.3	**−42.1 (−60.8, −23.3)**
Broccoli	21.4	68.9	**−47.5 (−61.6, −33.6)**
Rice	48.1	100.4	**−52.3 (−69.6, −35.1)**
Apple	52.4	87.3	**−34.8 (−58.1, −11.4)**
Milk	120.5	196.0	**−75.6 (−129.0, −22.1)**
**Snack Meal Offerings (true grams–recalled grams)**			
Crackers	14.2	12.6	1.6 (−1.9, 5.1)
Cheese	15.4	36.1	**−20.6 (−30.1, −4.6)**
**Dinner Meal Offerings (true grams–recalled grams)**			
Pasta	94.4	129.1	−34.7 (−73.4, 3.9)
Salad	16.9	30.8	**−13.9 (−22.3, −5.4)**
Garlic Bread	13.9	28.4	**−14.5 (−6.7, −3.8)**
Fruit Salad	59.9	80.9	−20.9 (−47.3, 5.3)
Cookie	14.5	10.5	3.9 (1.2, 6.7)
Juice Box	149.8	180.1	−30.3 (−142.2, 81.5)
Milk	60.0	151.2	**−91.2 (−136.9, −45.6)**
Water	50.9	108.1	**−57.1 (−110.2, −4.2)**

Bolded values represent significance at *p* < 0.05, confidence interval does not include 1.
